# Gastric Plexiform Fibromyxoma Arising in the Cardia in an Adolescent Male: A Rare Tumor with an Unusual Location

**DOI:** 10.1155/2020/9037960

**Published:** 2020-11-05

**Authors:** Awrad Nasralla, Mufeed Alwabari, Osama Alsaif, Samir S. Amr

**Affiliations:** ^1^Department of Surgery, King Fahad Specialist Hospital-Dammam, Saudi Arabia; ^2^Department of Pathology and Laboratory Medicine, King Fahad Specialist Hospital-Dammam, Saudi Arabia

## Abstract

Plexiform fibromyxoma of the stomach, also known as plexiform angiomyxoid myofibroblastic tumor, is a rare benign gastric mesenchymal tumor, first described in 2007, which usually arises in the gastric antrum and affects adults. Few cases have been reported in children and adolescents. It can present with different clinical manifestations including abdominal pain, dyspepsia, hematemesis, and vomiting. Preoperatively, this tumor is usually diagnosed as gastrointestinal stromal tumor (GIST), and the correct diagnosis is made only after histopathological examination following surgical resection. Most cases were reported from East Asia (China, Japan, and Korea), North America, and Europe. We report herein a unique case of plexiform fibromyxoma, the first to be reported from the Middle East, arising in the cardia of the stomach in a 16-year-old adolescent male, with a brief review of the literature.

## 1. Introduction

Plexiform fibromyxoma (PF) is a rare benign gastric mesenchymal tumor, described for the first time in 2007 by Takahashi et al., who named it plexiform angiomyxoid myofibroblastic tumor of the stomach [1]. It affects adults mainly, with a few cases observed in children and adolescents [2, 3]. It is usually located at the antrum, with occasional cases found in the body of the stomach or rarely presenting primarily in the duodenum [4, 5], the esophagus [6], the jejunum [7], the colon [8], and the gallbladder [9]. It has variable clinical presentations, including abdominal pain, dyspepsia, and vomiting. Occasionally, this tumor presents with bleeding, obstruction, and perforation. In addition, it could be found incidentally during endoscopy or radiological images [2, 3, 10]. In a retrospective histologic review of approximately 4200 GISTs from 1970 to 1999, Miettinen et al. found ten cases and added two additional cases of benign gastric antral fibromyxoid tumors with a peculiar multinodular, plexiform pattern, and they proposed the name plexiform fibromyxoma. They pointed that this tumor is usually confused with the myxoid variant of GIST. Definite diagnosis is usually confirmed after surgical excision and histopathological examination of the specimen [10]. Treatment is surgical resection of the tumor; however, the type of surgery depends on the size and location of the tumor [3, 10]. Herein, we report the twelfth case of gastric plexiform fibromyxoma in the pediatric population with an unusual location in the cardia, a location reported for the first time for this tumor.

## 2. Case Presentation

A 16-year-old boy presented with a recent history of two episodes of hematemesis, without associated other gastrointestinal symptoms. Six months earlier, he started dieting to lose weight, and he lost around 20 kilograms. Past medical, surgical, and family histories were unremarkable. Initially, he was seen at a local hospital, and a computed tomography (CT) scan was done there. CT scan showed a submucosal gastric mass. The patient then was referred to our hospital for further management.

On physical examination, the patient was overweight, looking pale, but not in pain or distress. His vital signs were within normal limits. His abdomen was soft, not tender or distended, with no palpable masses. Laboratory investigations including hepatic and renal function tests, electrolytes, and coagulation profile were all within normal. However, complete blood count (CBC) revealed low hemoglobin values (8.2 grams per dL) which are most likely related to hematemesis.

**Figure 1:**
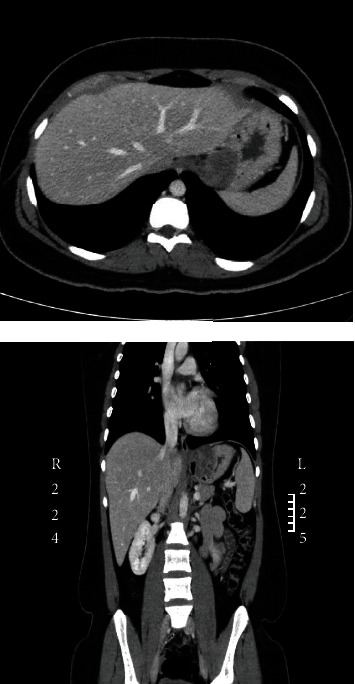
CT images showing a gastric mass. A 4.7 cm lobulated submucosal gastric mass at the gastric cardia near the gastroesophageal junction. The mass has an exophytic component abutting the left hemidiaphragm and left liver lobe.

Computed tomography (CT) scan revealed a lobulated submucosal gastric mass at the gastric cardia near the gastroesophageal junction measuring 4.7 × 4.3 × 4 cm. The mass had a predominantly low attenuation component with central gas component which could be due to an associated ulcer. Superiorly, the mass had an exophytic component abutting the left hemidiaphragm and near the inferior aspect of the left hepatic lobe (Figure 1). The remainder of the stomach was unremarkable. There was no gastric outlet obstruction. The small and large bowel loops were unremarkable, and no abdominal lymphadenopathy was noted. The location, radiological appearance, and lack of lymphadenopathy were suggestive of mesenchymal tumor, most likely gastrointestinal stromal tumor (GIST). The location was not typical for leiomyoma, and the heterogeneous attenuation makes schwannoma less likely. There were no thoracic, abdominal, or pelvic metastatic deposits. Correlation with endoscopy was recommended.

**Figure 2:**
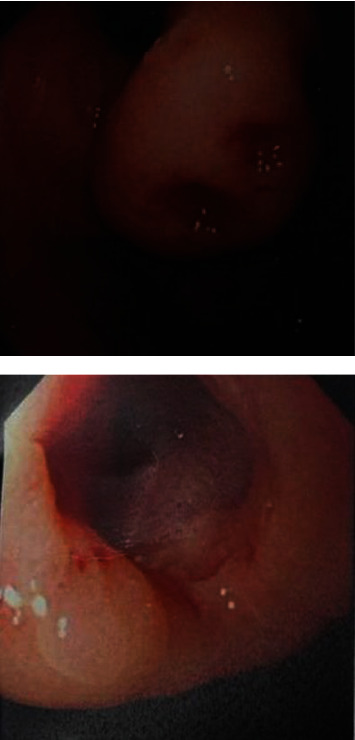
Upper gastrointestinal endoscopy revealed submucosal mass measuring 4.7 cm, located at the cardia, with deep ulcer.

Upper gastrointestinal (GI) endoscopy was done and showed normal esophagus, submucosal mass (5 cm) with deep ulcer at the cardia, and first and second parts of the duodenum were normal (Figure 2). In addition, endoscopic ultrasound (EUS) was done, and it demonstrated a submucosal mass at the cardia measuring 5 × 3 cm. It was oval in shape, heterogeneous, echogenic, soft, arising from muscularis propria, with no appreciable adjacent lymph nodes. These findings were suggestive of gastrointestinal stromal tumor (GIST).

The patient underwent laparotomy with wedge resection of the mass at the gastroesophageal junction, with primary closure of the stomach. The postoperative course was uneventful.

Pathological examination of the specimen, which was labelled as “gastroesophageal junction mass,” revealed a single mass covered by mucosa featuring three ulcerated areas; the largest measured 1.5 × 1 × 0.6 cm and the other small measuring 0.6 and 0.5 cm in diameter. The mass was serially sectioned revealing a single rubbery soft white homogenous well-circumscribed tumor with whorly appearance, measuring 5 × 5 × 4.5 cm. The ulcerated area showed necrotic material inside the cavity of the ulcer. On histopathological examination, sections showed a mesenchymal myxoid multinodular plexiform tumor arising within the muscularis propria that extended to the submucosa and reached into areas of the muscularis mucosae. The overlying gastric mucosa featured areas of ulceration covered by neutrophilic exudate with granulation tissue formation, and mixed inflammatory infiltrate was noted. Lymphoid follicles were seen in the lamina propria.

**Figure 3:**
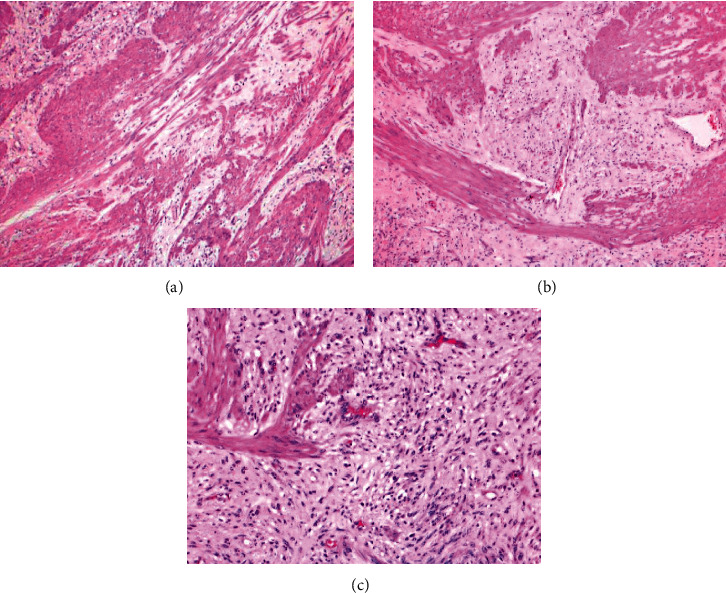
(a) Low-power magnification featuring bundles of smooth muscle fibers with intervening vascularized myxoid tumor, forming a plexiform pattern (H&E ×40). (b) Low-power magnification featuring myxoid vascularized areas alternating with cellular bundles of smooth muscle fibers (H&E ×40). (c) Spindle-shaped cells free of atypia or mitotic activity within myxoid stroma (H&E ×100).

**Figure 4:**
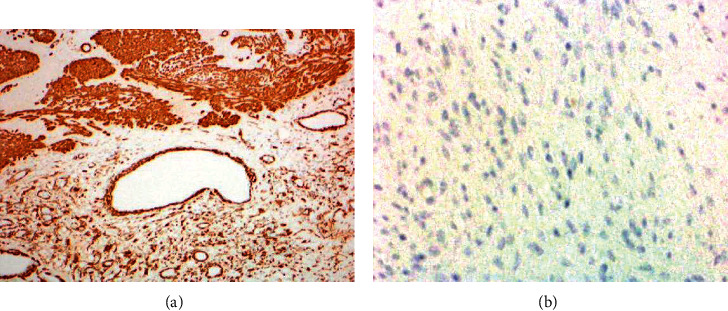
(a) Immunohistochemical stain for smooth muscle actin (SMA) featuring positive staining of normal muscle fibers of the stomach, walls of blood vessels, and spindle tumor cells. (b) Immunohistochemical stain for CD117 (C-Kit), a marker for GIST, featuring negative staining of spindle tumor cells.

The tumor was composed of nodules of spindly mesenchymal cells with elongated nuclei and conspicuous nucleoli and eosinophilic fibrillary cytoplasm, embedded within loose myxoid stroma with scattered capillaries seen. The tumor nodules were seen dissecting their way or surrounded or traversed by bundles of smooth muscle fibers derived from the muscularis propria resulting in a plexiform pattern (Figures 3(a)–3(c)). Immunohistochemical stains of the spindly tumor cells revealed positive staining for vimentin and smooth muscle actin and negative staining for CD117 and DOG-1 (GIST markers). Ki-67 proliferation marker was quite low at 2%. These histological and immunohistochemical findings confirmed this tumor as PF (Figures 4(a) and 4(b)).

One week postoperatively, upper GI contrast study revealed free flow of contrast along the whole segments of the esophagus and through the gastroesophageal junction down to the stomach with no evidence of contrast leakage. Diet was advanced as the patient tolerated. The patient was discharged home with analgesic, pantoprazole, and follow-up appointment in the clinic.

On follow-up visits at the clinic, he was doing well, tolerating diet, and did not have any episodes of hematemesis, abdominal pain, or reflux. Four months following surgery, upper GI endoscopy was done, and it demonstrated gastric esophageal reflux (GERD) with mucosal break > 5 mm above the Z line with sutures on the other site. The endoscopic examination of the stomach showed folded mucosa near the cardia, with normal appearance. A scar of recently healed ulcer was seen in the first part of the duodenum. Biopsy taken during the endoscopy showed chronic active follicular gastritis with *Helicobacter pylori*. The patient was started on proton pump inhibitor (PPI) drugs and treatment for *Helicobacter pylori*.

Three years following his surgery, he was doing well and tolerating diet. He did not have any episodes of hematemesis, abdominal pain, or reflex. His most recent upper GI endoscopy at 30 months postoperatively showed no tumor recurrence. We planned to follow up the patient every 6 months to ensure the absence of any recurrences.

## 3. Discussion

Since its initial description in 2007, this rare gastric neoplasm that was named initially as “plexiform angiomyxoid myofibroblastic tumor” (PAMT) [1] had been designated in the most recent classification of tumors of the digestive system by the World Health Organization (WHO) in 2010 as “plexiform fibromyxoma” (PF), and this is the preferred name assigned to this tumor in the current literature [11]. In a recent review, Fukazawa et al. collected 79 cases labelled as PAMT or PF and added one of their own. It is more common in adults than in children with a ratio of 7 : 1 and equal male to female predisposition [2]. More cases were reviewed in 2019 by Su et al., who collected a total of 121 cases, providing a more comprehensive updating of PF [3]. They stated that the age range of the patients was broad ranging from 5 to 81 years (mean age 43.17 ± 18.00 years, median age 46 years), with most patients middle aged, with a peak around 30-60 years. Their findings showed adult-to-child ratio to be 8 : 1, a figure close to that reported by Fukazawa et al. [2]. They found a slight preponderance in females, with male patients accounting for 43% and female patients for 57% of reported cases. They demonstrated that most cases were reported from East Asian countries, including China, Japan, and Korea (58 cases, 47.9% of reported cases). This is followed by North America (29 cases, 24.0%), Europe (26 cases, 21.5%), with a few cases from South East Asia, South Asia, and Africa. No cases were reported from the Middle East, thus making the current case the first to be reported from that region.

The most common location of PF is in the gastric antrum (95 cases, 79.2%); in some cases, the duodenal bulb is involved (22%). However, it is less common to be found at the gastric body (10 cases, 8.3%) and at the gastric fundus (4 cases, 3.3%) [3]. There is one reported pediatric case of PF located in the esophagus [5]. Our case is the first one to report the tumor at the gastric cardia near the gastroesophageal junction in the pediatric age group.

In addition to the current case, there had been eleven previously reported cases of PF in children below the age of 18 years (Table 1). Their ages ranged from 5 to 18 years. There was a predominance of females (8) over males (4), with male to female ratio of 1 : 2. The size of the tumors ranged from 3.2 to 17 cm in diameter (mean size was 7.3 cm). Eight cases were located in the gastric antrum, two cases in the gastric pylorus, one case in the gastric cardia (current case), and one case in the esophagus. Eight tumors were treated by partial gastrectomy; one by distal gastrectomy; two by tumor resection; and in one case, the modality of treatment was not stated [2, 5, 10, 12–17].

PF presented usually as a submucosal mass; however, it could involve any layer from the gastric serosa to the gastric mucosa. Finding of ulcer associated with tumor was not uncommon, and it explained the hematemesis our patient had. The size of the tumor varied from 1.5 cm up to 15 cm [2, 3].

Its diagnosis prior to surgery is difficult and is often confused with GIST based on findings on images and endoscopy. In a recent multicenter study of seven cases, Lai et al. had intraoperative frozen sections and/or EUS-FNA on six cases, and all of them were diagnosed preoperatively or intraoperatively as GIST. EUS-FNA material showed elongated spindle cells with streaming oval or elongated nuclei, features that can be seen in GIST [18]. PF can be misdiagnosed as GIST on frozen section due to its similarity to the myxoid variant of GIST. Immunostains are required to confirm the diagnosis of PF [10, 18]. In this case, gastric PF was not expected due to the age of the patient and the location of the tumor.

The role of CT scan in differentiating PF from GIST had been reported by Ikemura et al. They stated that PF showed a heterogeneous tumor in the gastric antrum which was drastically enhanced with contrast medium and consisted of a number of highly small nodules around the tumor rim. These CT findings reflect the characteristic growth pattern of PF and are claimed by the authors to be distinct enough to differentiate it from GIST [19].

Treatment is mainly surgical resection. The kind of surgical resection is usually determined by the size, the depth, and the location of the tumor. Surgical procedures include distal gastrectomy (most commonly reported surgery), partial gastrectomy, wedge resection, antrectomy, subtotal gastrectomy [2, 3], and endoscopic resection [20].

Among all the reported cases, PF exhibited a benign biological behavior, with low mitotic activity and no local recurrence or distant metastasis [7, 10]. In their review of 121 cases, Su et al. stated that 80 patients had a follow-up, with uneventful or alive duration ranging from 0.75 to 396 months, with no malignant change, local recurrence, or tumor-related mortality reported. However, they indicated that no consensus had been reached whether PF is actually a benign tumor, and no cases so far had confirmed that malignant change does not occur, so more longitudinal studies with sufficient number of cases are required. They pointed out that for the time being, PF should be considered a benign tumor [3].

## 4. Conclusion

Plexiform fibromyxoma, a rare mesenchymal gastric tumor, usually presents with nonspecific upper gastrointestinal symptoms. It is quite uncommon to be encountered in children with only ten cases on record. It is most commonly found in the gastric antrum but can be located anywhere in the stomach. We report the twelfth case in the pediatric age group, with the unusual location of the tumor at the gastric cardia near the gastroesophageal junction. Images and endoscopy can aid in assessing the location and the size of the tumor, which helps to decide the best surgical technique. However, the diagnosis is usually established after histopathological examination of the tumor following surgery. It seems that this tumor has no potential of recurrence or metastasis.

## Figures and Tables

**Table 1:** Reported cases of plexiform fibromyxoma in the pediatric age group.

No.	Author Year [Reference]	Age/gender	Location	Size (cm)	Symptoms	Treatment	Follow-up (months)
1	Miettinen et al. 2009 [10] Case 10	16/F	Gastric antrum, pylorus	10	Hematemesis	Partial gastrectomy	36
2	Miettinen et al. 2009 [10] Case 12	7/F	Gastric antrum, pylorus, duodenal bulb	15	Vomiting, diarrhea	Excision of tumor with gastric wall resection at the tumor attachment	Not stated
3	Duckworth et al. 2014 [6] Case 1	16/F	Esophagus and posterior mediastinum	3.2	Incidental finding on CT scan of the thorax	Tumor resection	14
4	Duckworth et al. 2014 [6] Case 2	11/F	Gastric pylorus	3.5	Severe iron deficiency anemia	Partial gastrectomy	15
5	Spans et al. 2016 [12]	18/F	Gastric antrum	4.5	Not stated	Not stated	Not stated
6	Morris et al. 2016 [13]	9/F	Gastric antrum	5	Abdominal pain, vomiting	Partial gastrectomy	6
7	Liang et al. 2017 [14]	11/M	Gastric pylorus	17	Abdominal pain	Partial gastrectomy	12
8	Szurian et al. 2017 [15]	16/F	Gastric antrum	6.5	Anemia	Partial gastrectomy	6
9	Djurić et al. 2018 [16]	14/M	Gastric antrum	5	Anemia	Partial gastrectomy	42
10	Fukazawa et al. 2019 [2]	14/F	Gastric antrum	5.5 cm	Abdominal pain, hematemesis	Partial gastrectomy	16
11	Li et al. 2019 [17]	5/M	Gastric antrum	8.5	Pale complexion	Distal gastrectomy	36
12	Nasralla et al. 2019 Current case	16/M	Gastric cardia near gastroesophageal junction	5	Anemia, hematemesis	Wedge resection of mass at GE junction	36
